# Captorhinid reptiles from the lower Permian Pedra de Fogo Formation, Piauí, Brazil: the earliest herbivorous tetrapods in Gondwana

**DOI:** 10.7717/peerj.8719

**Published:** 2020-03-06

**Authors:** Juan C. Cisneros, Kenneth Angielczyk, Christian F. Kammerer, Roger M.H. Smith, Jörg Fröbisch, Claudia A. Marsicano, Martha Richter

**Affiliations:** 1 Museu de Arqueologia e Paleontologia, Universidade Federal do Piauí, Teresina, Piauí, Brazil; 2 Negaunee Integrative Research Center, Field Museum of Natural History, Chicago, IL, USA; 3 North Carolina Museum of Natural Sciences, Raleigh, NC, USA; 4 Department of Karoo Palaeontology, Iziko South African Museum, Cape Town, Western Cape, South Africa; 5 Evolutionary Studies Institute, University of Witwatersrand, Johannesburg, Gauteng, South Africa; 6 Museum für Naturkunde, Leibniz-Institut für Evolutions- und Biodiversitätsforschung, Berlin, Germany; 7 Institut für Biologie, Humboldt Universität Berlin, Berlin, Germany; 8 Departamento de Ciencias Geológicas, FCEN, IDEAN-CONICET, Universidad de Buenos Aires, Buenos Aires, Argentina; 9 Earth Sciences Department, Natural History Museum, London, UK

**Keywords:** Reptilia, Captorhinidae, South America, Gondwana, Parnaíba basin, Cisuralian, Permian, Herbivory

## Abstract

The Pedra de Fogo Formation in the Parnaíba Basin of northeastern Brazil hosts a recently discovered lacustrine fauna and provides the only known record of the Captorhinidae in South America. Here, new captorhinid remains from this unit are described. Two partial mandibles, including one formerly ascribed to the genus *Captorhinus*, are here referred to *Captorhinikos* sp. a genus previously described from North America. The natural mould of a large mandible probably represents a new taxon within the captorhinid subclade Moradisaurinae, and a small skull roof is regarded as Captorhinidae indet. Captorhinids are generally considered to have been herbivores or omnivores. The Pedra de Fogo captorhinids likely played an important ecological role as primary consumers in the palaeoenvironment of this geological unit, which is also known for its extensive record of petrified forests. The new finds reinforce the close relationships between the continental faunas of palaeotropical western Gondwana and palaeoequatorial North America during the Cisuralian.

## Introduction

The lower Permian (Cisuralian) was a time of major diversification for herbivorous tetrapods. Although tetrapod herbivores first appeared in the late Carboniferous (diadectomorphs and edaphosaurid synapsids), these groups increased in richness in the Permian and were joined by a novel array of herbivorous reptile groups (Sues & Reisz, 1998). Among these groups were the Captorhinidae, a very successful clade of small (~4 cm total skull length) to large (~45 cm total skull length) reptiles that flourished throughout most of the Permian (O’Keefe et al., 2005; Brocklehurst, 2017; Romano, Brocklehurst & Fröbisch, 2017; Romano & Rubidge, 2019). Captorhinids evolved multiple tooth rows for dental occlusion that facilitated oral processing of low- to high-fibre food items (Hotton, Olson & Beerbower, 1997; Sues & Reisz, 1998; Reisz & Sues, 2000; Reisz, 2006). Their earliest records are from North America in the late Carboniferous (Müller & Reisz, 2005) and they became conspicuous components of North American terrestrial ecosystems during the Cisuralian. Their remains are also found in Guadalupian and Lopingian rocks in Spain (Liebrecht et al., 2017), Germany (Sues & Munk, 1996), Russia (Vjuschkov & Tchudinov, 1957; Ivakhnenko, 1990), China (Reisz et al., 2011), Morocco (Jalil & Dutuit, 1996), Niger (De Ricqlès & Taquet, 1982), South Africa (Modesto & Smith, 2001), Zambia (Gow, 2000), Zimbabwe (Gaffney & Mckenna, 1979), and India (Kutty, 1972). Despite their otherwise broad geographic distribution, however, until recently captorhinids were unknown in the Permo-Carboniferous record of South America.

**Figure 1:**
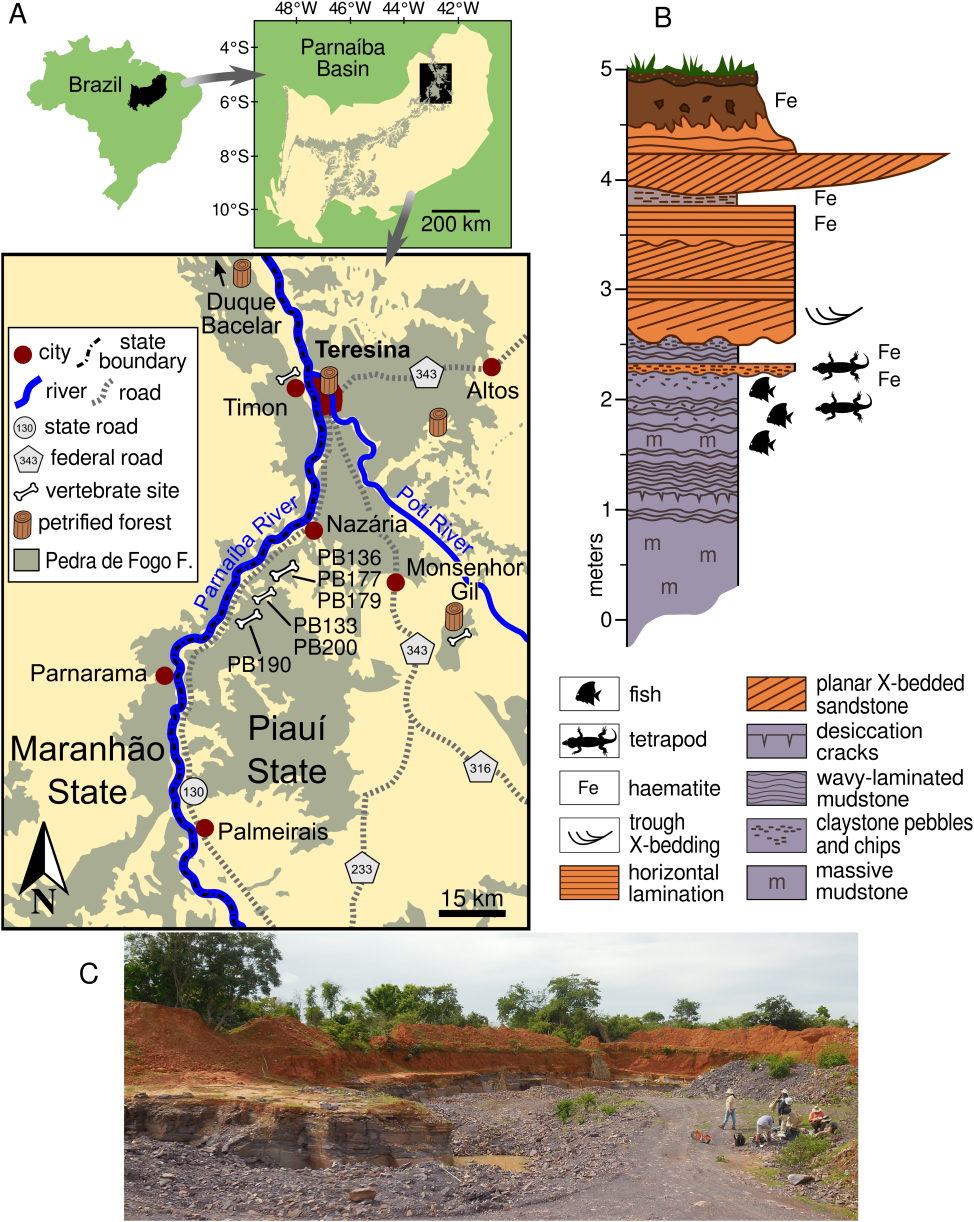
Study area and geology. (A) Location of the Pedra de Fogo fossil sites in the Teresina area within the Parnaíba Basin. (B) Sedimentology of the area, based on PB133 and PB200 quarries, both located in Nazária. (C) Overview of the PB190 quarry in Palmeirais.

The Pedra de Fogo Formation of the Parnaíba Basin, northeastern Brazil, has long been known for its rich palaeobotanical record (Lisboa, 1914; Caldas et al., 1989; Santos & De Carvalho, 2004; Dias-Brito et al., 2009; Iannuzzi et al., 2018) and aquatic vertebrates (Silva Santos, 1990, 1994; Figueroa & Gallo, 2017). The latter were mainly collected from the central portion of the basin in strata attributed to fluvio-marine environments, with the large temnospondyl *Prionosuchus plummeri* being the only tetrapod known (Price, 1948; Cox & Hutchinson, 1991). In recent years, a continental fauna was discovered in Pedra de Fogo exposures in quarries around the city of Teresina (Fig. 1A) in the northeastern portion of the basin (Cisneros et al., 2015; Iannuzzi et al., 2018). This area has produced a rich lacustrine biota and a neighbouring petrified forest that, among a variety of tetrapods, includes the first captorhinid record in South America, which was initially referred to *Captorhinus aguti* (Cisneros et al., 2015). Ongoing collecting efforts have yielded additional captorhinid specimens from this area, including a second, larger taxon. Here we describe the Pedra de Fogo captorhinids and discuss their evolutionary, ecological and biogeographical implications.

## Materials and Methods

Specimen MAP PV014 was prepared using standard mechanical methods. Positive silicone casts were made of the natural moulds MAP PV014 and MAP PV664. A cladistic analysis was carried out using an expanded version of the character-taxon matrix from Modesto, Scott & Reisz (2018). See Supporting Information for the data matrix, new characters included in the analysis, and coding amendments. The character-taxon matrix was compiled in Mesquite 3.2 (Maddison & Maddison, 2018). The phylogenetic analyses were performed using TNT version 1.5 (Goloboff & Catalano, 2016). Two analyses were run. In the first, all characters were treated as unordered and unweighted. Symmetric resampling support values were calculated from 5,000 replicates. In the second analysis the ‘implied weighting’ option (weighting against homoplasy) was employed (weight function *k* = 3). In both analyses, ambiguously supported branches were collapsed (‘collapsing rule 1’), and the implicit enumeration algorithm was used for tree searching *Captorhinus kierani* was coded for the cladistic analysis based on the description of DeBraga, Bevitt & Reisz (2019). Tooth nomenclature follows Smith & Dodson (2003). Fieldwork was authorised by Agencia Nacional de Mineração through collecting request 000.180/2016.

### Study area and geological setting

The vertebrate sites under consideration are located in the northern portion of the Parnaíba Basin, within the municipalities of Nazária and Palmeirais in the state of Piauí, some 60 km south-west of Teresina, the state capital (Fig. 1A). All specimens were recovered at quarries along the PI-130 highway, where silicified claystone blocks are extracted for use as construction material in Teresina and neighbouring cities. The Pedra de Fogo exposures that produce the vertebrates under consideration are located only a few kilometres away from petrified forests, some containing trees in life position (Caldas et al., 1989; Da Conceição et al., 2016; Da Conceição, Cisneros & Iannuzzi, 2016), and microbialites (Fairchild, Rohn & Dias-Brito, 2015).

The Silurian-to-Jurassic-aged Parnaíba Basin is an intracratonic crustal sag that affected some 600.000 km^2^ of north-central Gondwana, the cause of which is uncertain but likely linked to thermally-induced downwarping (Daly et al., 2014). During the Permian, when the Pedra de Fogo Formation was deposited, the basin was situated within the southern tropics (Linol et al., 2016) under a warm semi-arid climate that became increasingly dry in the Triassic (Iannuzzi et al., 2018).

The Pedra de Fogo Formation sediments accumulated on extensive lowland plains that were affected by differential rates of subsidence (Daly et al., 2018), resulting in a switch between lacustrine wetlands and fluvial floodplain-dominated sedimentation. This repeated, tectonically-induced lowering of base level is evidenced in the Pedra de Fogo Formation succession by stacked first order fining-upward sequences up to 50 m thick comprising fluvial channel sandstones, followed by fine-grained sandstones/mudstones, oolitic and concretionary limestone, and finally fossil-bearing laminated mudrock with stromatolitic limestone lenses (Aguiar, 1971). At times, when lake levels dropped, large scale desiccation cracks with distinctive tepee structures (Kendall & Warren, 1987) disrupted the sub-aerially exposed carbonate mudflats, leaving behind the distinctive ‘silex’ layer of this formation in the southern and western parts of the basin (Araújo et al., 2016).

Recent work on the Pedra de Fogo Formation near the eastern margin of the Parnaíba Basin, where the new captorhinids were found, suggests a more terrestrial depositional environment in that area, with marginal lacustrine facies still present but with more floodplain and fluvial channel deposits than in the southern and western parts of the basin (Cisneros et al., 2015). The building stone quarries in the Nazária/Palmeirais area (Fig. 1) all exploit the same interval of well-consolidated massive and horizontally-laminated mudrock (see 0–2.5 m on Fig. 1B), which has been mildly metamorphosed such that it can be readily cleaved into brick-sized blocks. The fossils were found in loose blocks close to the quarry face, so that it was possible to identify the exact horizon from which they had been excavated by quarry workers. The captorhinids from the quarry site PB190 (Fig. 1C) were preserved in a massively-bedded light-brown mudstone containing randomly orientated claystone chips within the upper wavy-laminated part of the lacustrine facies. Further along strike, this facies displays sand-filled polygonal mudcracks associated with algal mounds and gypsum blade casts. In places, the fossiliferous interval has been incised by a series of 5–10 m wide channels filled with climbing ripple cross-laminated fine-grained sandstone. Fossil fish remains occur in the same facies as the captorhinids, mainly as ‘fish hash’ lenses lining the axes of the scour troughs. These lenses are packed with isolated scales, teeth, and disarticulated skeletal elements, as well as intact scale-bearing spiral coprolites.

The massive and thinly-laminated mudrocks are interpreted as having accumulated in a deeper lacustrine setting (Araújo et al., 2016). The massively-bedded mudstone with floating claystone chips in which the large captorhinid jaw was preserved likely resulted from density underflow following a pulse of sediment being dumped into the lake margin by flooding rivers. The scoured channels filled with climbing ripple lamination attest to the rapid deceleration of sediment-laden floodwaters as they entered the lake margin. It is likely that these flood discharges were able to transport and concentrate the disarticulated fish and tetrapod bones into scour troughs eroded into the offshore lakebed. Similar fluvially-incised channels filled with rapidly deposited siltstone have been documented on the floor of the modern Lake St. Lucia, an estuarine-influenced lake/wetland system on the north eastern coastal plain of South Africa (Botha, Haldorsen & Porat, 2013).

## Systematic Palaeontology

Eureptilia Olson, 1947

Captorhinidae Case, 1911

*Captorhinikos* Olson, 1954

*Captorhinikos* sp.

**Referred Specimens.** MAP PV014, natural mould of right hemimandible. MAP PV855, right dentary.

**Horizon and Locality.** Pedra de Fogo Formation, Cisuralian. MAP PV014 was collected at quarry PB179 (Fig. 1A), in the municipality of Nazária. MAP PV855 was collected at quarry PB190 (Figs. 1A and 1C), in the municipality of Palmeirais. Both localities are in the state of Piauí, Brazil.

**Figure 2:**
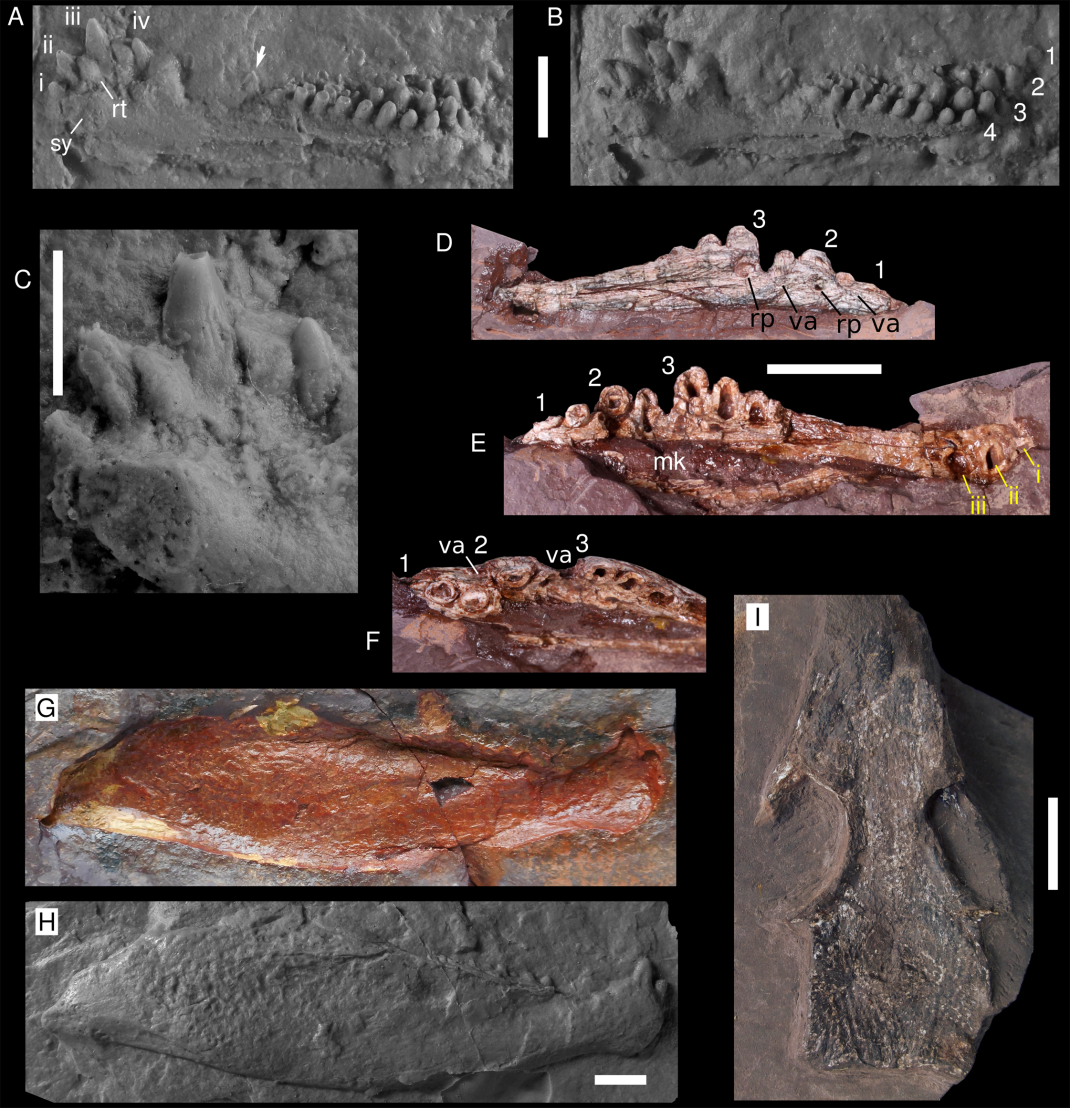
Captorhinids from the Pedra de Fogo Formation. (A–C) *Captorhinikos* sp, MAP PV014, silicon positive cast in dorsomedial view: (A) light from the right, (B) light from the left, (C) detail of incisiforms (photograph by Stephanie Ware). (D–F) *Captorhinikos* sp., MAP PV855, right dentary in (D) medial, (E) lateral, (F) occlusal views. (G–H) Moradisaurinae indet., MAP PV664, right hemimandible exposed in lateral aspect: (G) natural mould (reversed), (H) positive silicon cast. (I) Captorhinidae indet., MAP PV015, skull roof in dorsal view. Abbreviations: mk, Meckelian cavity; rp, resorption pit; replacement tooth; sy, symphysis; va, vacant alveolus. Arrows indicate resorption pits. 1–4, tooth rows (from labialmost to lingualmost); i–iv, tooth positions. Scale bars equal 10 mm, except for F (5 mm).

**Description.** MAP PV014 is a natural impression of the anterior portion of a right hemimandible (Figs. 2A and 2B; also see Cisneros et al., 2015) that can be studied from its cast, mostly consisting of the tooth-bearing area of the dentary, exposed in lingual (slightly occlusal) view. The dentary contribution to the symphysis is preserved and well exposed in medial view (Fig. 2C). The symphysis is ovoid and obliquely aligned in relation to the anteroposterior axis of the mandible. No traces of the splenial bone can be recognised, but an elongated, empty space that extends anteriorly below the tooth-bearing region of the symphysis likely represents the area of the dentary that accommodated the lateral surface of the missing splenial.

The anteriormost teeth are mainly conical and incisiform, and are arranged a single row consisting of at least four teeth. The first tooth is the smallest incisiform and it is strongly inclined anteriorly (~45°). The subsequent teeth become larger and progressively less inclined. The third incisiform is the largest tooth in the single row, being more than twice the size of the first. One additional incisiform is located medially between the second and the third teeth. This tooth is approximately the same size as the second incisiform, and judging by its lingual placement in relation to the single row, it likely represents a replacement tooth. Delicate fluting is present on the incisiforms (Fig. 2C). These striations cover most of the tooth crown, gradually becoming fainter toward the tip. The tooth-bearing region between the incisiforms and the posterior multiple rows is damaged. Only one damaged tooth (see arrow in Figs. 2A and 2B) is visible here, immediately anterior to the start of the multiple tooth rowed region. Posteriorly, four rows of teeth are present. Only one tooth is visible from the first (labialmost) row. The second row has at least four teeth. The third row bears nine teeth. The fourth (lingualmost) row possesses at least eight teeth. Teeth tend to increase in size posteriorly in the tooth-rows.

**Figure 3:**
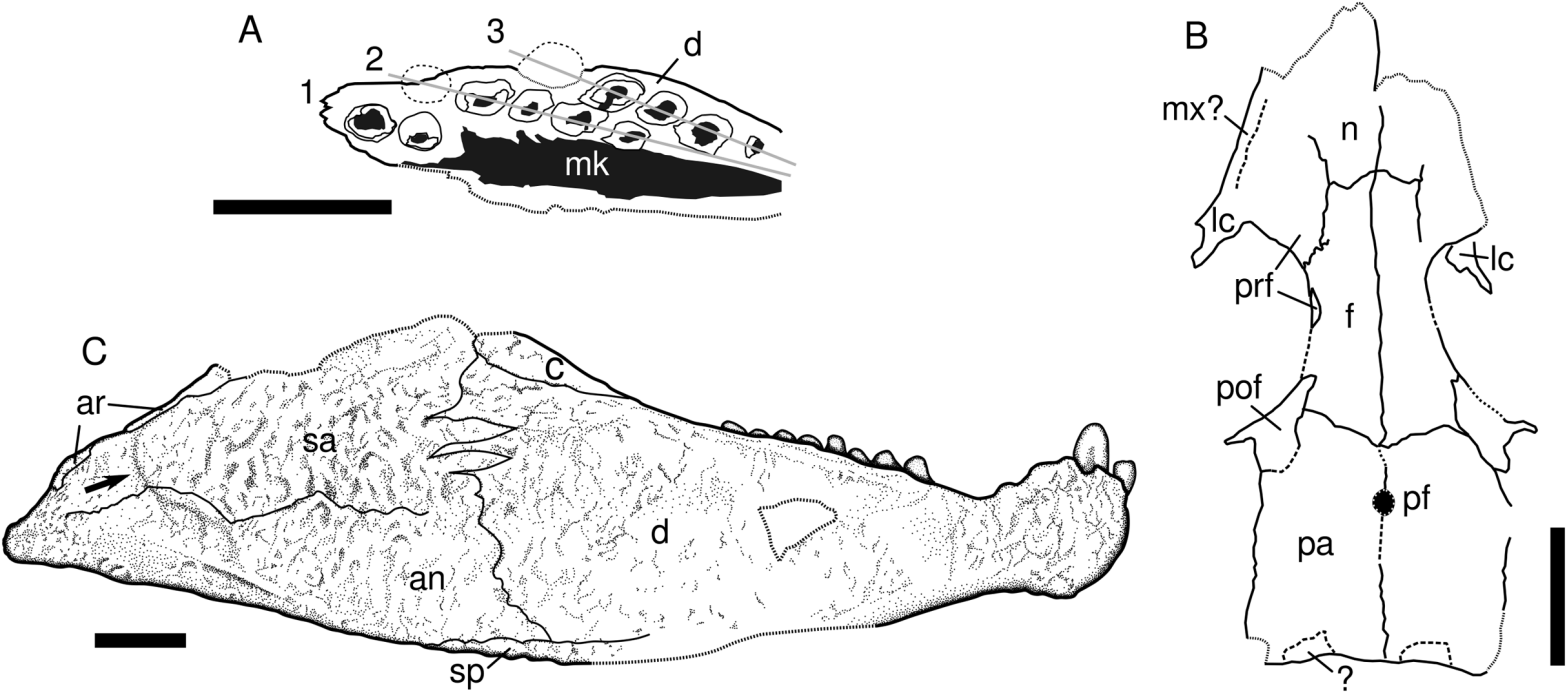
Captorhinids from the Pedra de Fogo Formation, interpretative drawings. (A) *Captorhinikos* sp., MAP PV855, right hemimandible in occlusal view. (B) Captorhinidae indet. MAP PV015, skull roof in dorsal view, interpretative drawing. (C) Moradisaurinae indet., MAP PV664, right hemimandible in lateral aspect. Scale bars equal 10 mm. Abbreviations: an, angular; ar, articular; c, coronoid; d, dentary; f, frontal; 1–3, tooth-rows; lc, lacrimal; mk, Meckelian cavity; mx, maxilla; n, nasal; pa, parietal; pf, pineal foramen; pof, postfrontal; prf, prefrontal; sa, surangular; sp, splenial. Arrow represents sigmoidal step at posterior end of the lateral mandibular shelf.

MAP PV855 is a partially exposed right dentary that preserves at least three tooth rows (Figs. 2C–2E and 3A). The third tooth in the anterior single row is the largest. The labial surface is damaged, partially exposing the cavity for the Meckelian cartilage. The first (labialmost) tooth row is very damaged and only two teeth can be recognised. The second tooth row bears at least five tooth positions, the posteriormost one being lost but recognised due to its vacant alveolus. The third tooth row also preserves at least five tooth positions, the fifth tooth being evinced by a vacant alveolus. All the teeth in the second and third rows are broken and exposed in parasagittal section. These rows are straight and convergent (radiating pattern, distinguishing them from all other moradisaurines sensu Modesto, Lamb & Reisz (2014)) (Fig. 3A). The teeth increase in size posteriorly, in contrast with *Captorhinus aguti*. The labial surface is relatively well preserved and shows four events of tooth replacement. Two resorption pits, and two vacant alveoli (replacement pits) are placed at the distal end of each tooth row on the lingual surface.

**Remarks.** MAP PV014 was previously referred to *Captorhinus aguti* by Cisneros et al. (2015). Further cleaning of the natural mould and the production of a silicone positive has permitted reidentification as *Captorhinikos* sp. Both MAP PV014 and MAP PV855 are similar to *Captorhinikos valensis* in having posterior tooth-rows arranged in a radiating pattern, with teeth increasing in size distally. Both jaws also share with the genus *Captorhinikos* the presence of bulbous teeth with a conical, vertical apex. The third incisor tooth is the largest in the anterior portion of the dentary, a feature also observed in *Captorhinikos valensis* (LeBlanc et al., 2015). In addition, the first teeth in the multiple tooth rows are much smaller than the last one in the single row, as is the case in *Captorhinikos valensis* (LeBlanc et al., 2015). MAP PV014, however, differs from *Captorhinikos valensis* in the presence of fluting on the dentition. This is a distinctive trait that has not been reported in other captorhinids, with the exception of the recently described *Opisthodontosaurus* from North America (Reisz et al., 2015), and may indicate the presence of plicidentine. In *Opisthodontosaurus*, fluting is very strong and concentrated on the apical regions of the teeth. In the Pedra de Fogo fossil, by contrast, the striae are more subtle, present at the base of the teeth and absent on the apices of the crowns. Unfortunately, damage in MAP PV855 precludes assessing the presence of fluting in the incisiforms of this specimen. Although the fluting observed in the incisors of MAP PV014 is very weak, it could indicate that the Pedra de Fogo material represents a new species of *Captorhinikos*. Confirmation of this hypothesis requires more complete specimens that preserve more of the mandible or skull. For these reasons, we currently refer MAP PV014 to *Captorhinikos* sp. Both dentaries display clear tooth replacement events, taking place between the incisors of MAP PV014 and in the multiple-rowed region of MAP PV855. The pattern of substitution along the lingual surface of the dentary is consistent with the model described for *Captorhinikos valensis* and *Captorhinus* spp. (LeBlanc et al., 2015; LeBlanc & Reisz, 2015).

Moradisaurinae De Ricqlès & Taquet, 1982

Gen et sp. indet.

**Referred Specimen.** MAP PV664, a right hemimandible (natural mould).

**Horizon and Locality.** Pedra de Fogo Formation, Cisuralian. Collected at quarry PB190 (Figs. 1A and 1C), in the Municipality of Palmeirais, Piauí, Brazil.

**Description.** The fossil consists of a right mandibular ramus, preserved as a natural mould in silicified claystone (Figs. 2G and 2H). This description is mostly based on the positive silicone cast and the sutures are tentative (Fig. 3C). The nearly complete jaw is preserved in lateral view. It is of considerable size (~111 mm length and ~32 mm deep), being larger than most early Permian captorhinid species but comparable to that of *Reiszorhinus olsoni* (Sumida et al., 2010). Its surface is covered by a distinct ornamentation formed by ridges that border irregularly-shaped depressions. This sculpturing pattern is evident on the anteriormost area of the dentary. It gradually thins posteriorly, becoming faint below the post-incisiform teeth. The ornamentation is more prominent on the posterior portion of the dentary, and is most strongly developed on the angular and surangular bones, similar to the condition seen in *Labidosaurikos*. The sculpturing becomes subtle again at the posterior end of the jaw, below the articular bone. The less-ornamented area of the angular and surangular is separated from the coarsely-sculptured area of the bones by an oblique, sigmoidal step, which represents the posterior end of the lateral mandibular shelf (see arrow in Fig. 3C). This step is a feature shared with *Moradisaurus* from Niger (Modesto et al., 2019: fig. 2A) and *Labidosaurikos* from the USA (Dodick & Modesto, 1995: text-figure 3A), although the lateral mandibular shelf in MAP PV664 is less developed than in those taxa.

The dentary forms the anterior portion of the jaw in lateral view. Its contact with the angular is represented by a nearly vertical suture located at the anteroposterior mid-length of the jaw. The dentary-surangular contact is present as an interdigitating suture ventrally that becomes straighter and angles weakly anterodorsally as it approaches the base of the coronoid. The most remarkable feature of this mandible is the bulbous shape of the symphysis, which is emphasised by a dorsoventral constriction located immediately posterior to it. A small part of the splenial is exposed in lateral view, mainly as a thin sheet below the angular and dentary bones. The full anterior extent of this bone cannot be assessed due to damage in the area.

Two prominent incisiforms are present on the dentary. The first one has a mostly cylindrical outline, and is slightly inclined anteriorly, but to a much lesser degree than in taxa such as *Captorhinikos valensis* or *Captorhinus* spp. It is followed by a second, vertical tooth that is more than twice the height of the first, a condition shared with *Labidosaurikos* (Dodick & Modesto, 1995). The larger incisiform bears a vertical depression on its labial aspect, but it is not clear if this is a natural feature or a taphonomic artefact. The dorsal margin of the dentary immediately posterior to the second tooth shows two sinuosities that could be interpreted as vacant alveoli that hosted two smaller, missing teeth. Posterior to this area the dorsal edge of the dentary is concave, followed by a straight, posterodorsally directed edge that shows no trace of dentition for nearly 10 mm and appears to be a genuine diastema. The dentition reappears posteriorly as a row of eight teeth. These are much smaller than either of the two incisiforms and are primarily bulbous, with their mesiodistal length in basal section being equal to or larger than their height. The teeth that occupy the third and fifth positions appear to be more labially placed than the others in the row. The more lingual teeth could either belong to a second, inner tooth-row, represent replacement teeth, or simply be teeth that are irregularly aligned.

Captorhinidae Case, 1911

Gen. et sp. indet.

**Referred Specimens.** MAP PV015, a skull roof.

**Horizon and Locality.** MAP PV015 was collected at quarry PB136 (Fig. 1A), in the Municipality of Nazária, Piauí, Brazil.

**Description.** The specimen consists of a small (~50 mm long) weathered skull roof, exposed in dorsal view (Figs. 2I and 3B). The bones display a honeycomb-like ornamentation, with shallow pits and ridges, that is characteristic of various captorhinids (Modesto, Scott & Reisz, 2018). The nasals are only partially preserved, and lack their anterior portions. Both prefrontals are also missing their anterior ends. The lateral contact between the prefrontal and the lacrimal is damaged on both sides and cannot be confidently assessed. The posterior border of the prefrontal and lacrimal contribute to half of the anterior rim of the orbit, but the exact extent of their contributions cannot be confirmed due to the damaged contact. A small elongated bone seen along the edge of the left orbit might represent the posterior process of the prefrontal. Most of the lacrimal has been weathered, being better preserved posteriorly, where the bone contributes to the anteroventral margin of the orbit. A step that runs longitudinally along the left border of the skull likely represents the contact between the worn lacrimal and the remaining maxilla. The frontal is an elongated, approximately quadrangular bone that is wider at its posterior border and narrows gradually towards its anterior border. Assuming that the prefrontal has a long posterior process, the frontal contribution to the dorsal margin of the orbit is rather restricted. The posterior contact of both frontals with the parietals is difficult to discern but it appears to be oblique. The postfrontal is a sub-triangular bone that makes a large contribution to the edge of the orbit. Medially, it contacts the frontal and the parietal. The nearly rectangular parietal is the largest bone in the skull roof and it covers most of the area posterior to the orbits. The pineal foramen could not be confidently traced. However, an area located anterior to the midlength of the parietals, where the bone is absent, is the probable location for this structure, as it is in most captorhinids. Small, transversally-elongated bones at the edge of the occiput may represent either the supratemporals or the postparietals, however, they are too damaged to confidently assess their identity. The occipital margin of the skull roof is weakly embayed medially.

**Remarks.** The medially embayed occipital edge of the skull roof is a condition recorded in *Captorhinus*, *Labidosaurus*, *Labidosauriscus*, and *Labidosaurikos* (Dodick & Modesto, 1995; Modesto, 1998; Modesto et al. 2007; Modesto, Scott & Reisz, 2018). The moderate, honeycomb-like ornamentation pattern seen in MAP PV015 is a primitive feature for Captorhinidae (Modesto, Scott & Reisz, 2018). This character is absent in the larger moradisaurines, which possess a derived, thicker cranial sculpturing featuring large, irregularly placed pits. It also excludes the recently described captorhinids *Opisthodontosaurus* (Reisz et al., 2015) and *Labidosauriscus* (Modesto, Scott & Reisz, 2018), both of which have a more subtle sculpturing with tiny pits and furrows but no ridges. The combination of sculpturing and the occipital configuration seen in MAP PV015 is only known in the genus *Captorhinus*. However, the relevant areas in the skull roof of *Captorhinikos* are not known (Jung, 2018; Modesto, Scott & Reisz, 2018), and MAP PV015 was recovered only 400 m from a *Captorhinikos* sp. jaw (MAP PV014) in the same stratigraphic layer. At present, this fossil can only be referred as an indeterminate captorhinid.

## Phylogenetic Analysis

**Figure 4:**
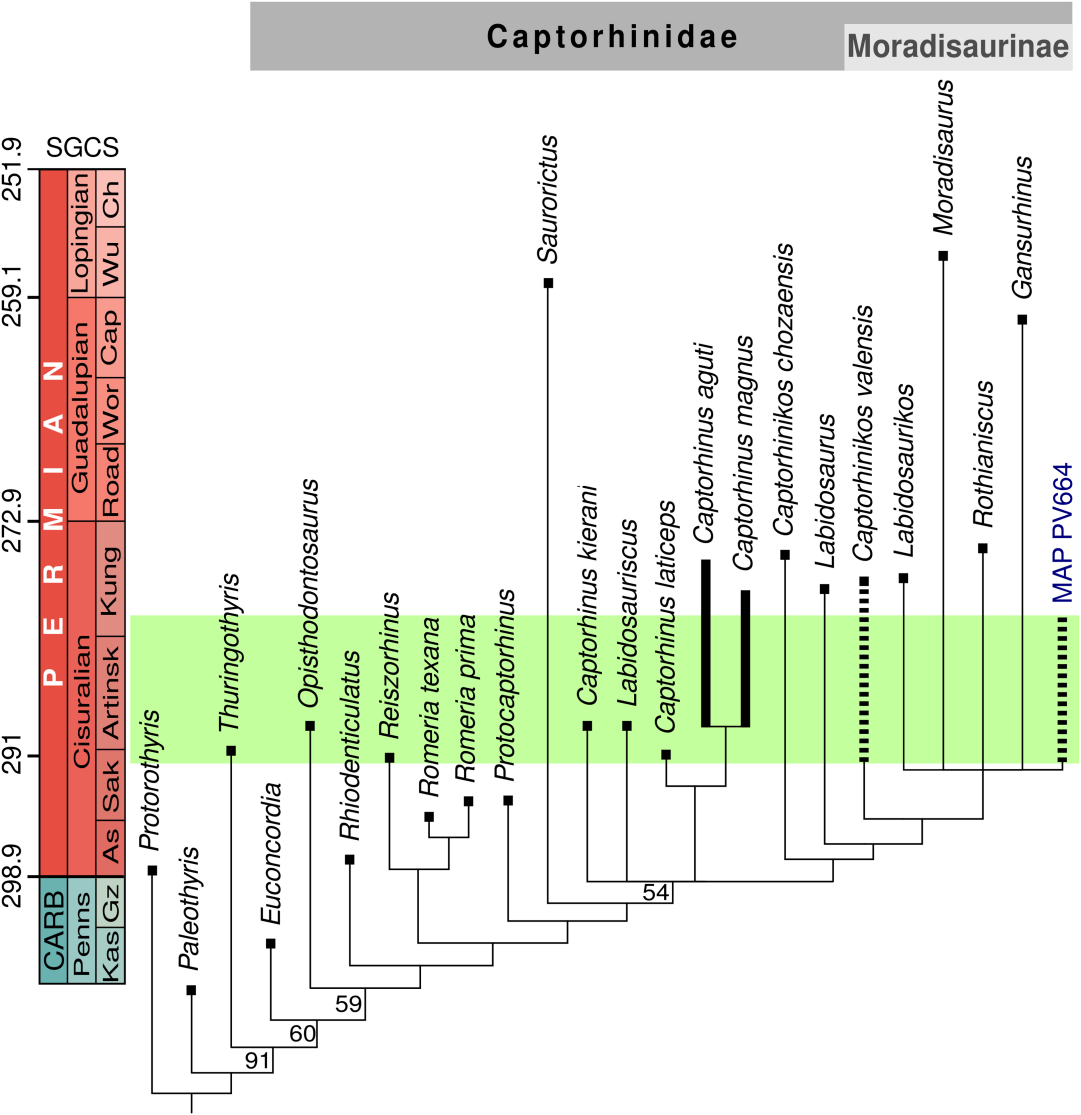
Stratigraphically-calibrated strict consensus cladogram showing the relationships of MAP PV664. Obtained from unweighted analysis (nine most-parsimonious trees, best score = 179). Resampling values (>50%) provided at the base of the nodes. Identical topology obtained from implied weighting analysis (four trees, best score = 15.87143). The green box represents the probable age of the Pedra de Fogo Formation.

The unweighted cladistic analysis found nine most-parsimonious trees (length 179 steps). The analysis using implied weighting recovered only four most-parsimonious trees (length 15.87143). The strict consensus tree generated by both analyses is identical (Fig. 4) and it has a generally similar topology to other recent analyses of captorhinid phylogeny (LeBlanc et al., 2015; Reisz et al., 2015; Liebrecht et al., 2017; Modesto, Scott & Reisz, 2018; Modesto et al., 2019). The main differences between our results and the previous ones can be explained in part by coding changes (both to characters and character states), but are mostly due to the inclusion of two new operational taxonomic units, *Captorhinus kierani* and MAP PV664. The species *Captorhinus kierani* was found to fall outside the genus *Captorhinus sensu stricto*, in a polytomy with a clade formed by the other three species of this genus, the recently described *Labidosauriscus*, and the Moradisaurinae. On the other hand, *Captorhinikos valensis* falls within Moradisaurinae, in a placement that does not differ from the results of the aforementioned analyses. The phylogenetic analysis places specimen MAP PV664 within Moradisaurinae, in a polytomy with *Rothianiscus* and *Labidosaurikos* from the United States, *Moradisaurus* from Niger, and *Gansurhinus* from China. That MAP PV664 appears as a moradisaurine captorhinid may not be surprising, considering the large size of this fossil, which is characteristic of several members of this clade. However, the deeper jaw of MAP PV664 contrasts with the shallower jaws of other moradisaurines such as *Moradisaurus* and *Labidosaurikos*, although this feature could be a taphonomic artefact, as in a *Moradisaurus* specimen described by Modesto et al. (2019: fig.1E, F) The incipient lateral shelf in MAP PV664 also contrasts with the prominent shelf that is present in these forms. The character-state that supports the placement of MAP PV664 within this polytomy is the reduction of tooth stations to 13 or less [6:3]. New characters added to this study, mainly the presence of a diastema [78:1], jaw sculpturing more developed posteriorly [79:2] and a sigmoidal step posterior to the lateral shelf [80:1] relate MAP PV664 to *Labidosaurikos*, or place it in a trichotomy with the *Labidosaurikos* and *Gansurhinus*, in the most-parsimonious trees. Support for MAP PV664 in the resulting polytomy in the consensus tree, however, is weak, and it should be regarded as tentative. More material of this morphotype, particularly from the cranium, will be required before it can be robustly placed in captorhinid phylogeny.

## Discussion

The age of the Pedra de Fogo Formation fauna has long been the subject of debate (Price, 1948; Cox & Hutchinson, 1991; Dino, Antonioli & Braz, 2002). It was recently considered to be of Kungurian age by Cisneros et al. (2015; also see Iannuzzi et al., 2018) based on correlation with both the Irati Formation in the Paraná Basin of southern Brazil and the Chemnitz petrified forest of Germany. The Pedra de Fogo chondrichthyans *Taquaralodus albuquerquei* and *Itapyrodus punctatus* are shared with the Irati Formation, a unit which has a U–Pb radioisotopic date of 278.4 ± 2.2 Ma (Santos et al., 2006). Other vertebrates known in the Pedra de Fogo are either endemic to the unit or have a stratigraphic range so extensive as to be useless as index fossils. For example the chondrichthyan genera *Sphenacanthus* and *Glikmanius* (Silva Santos, 1946; Figueroa & Gallo, 2017) are known elsewhere from the Carboniferous to the mid-late Permian (Pauliv, Dias & Sedor, 2012; Figueroa & Gallo, 2017). On the other hand, the rich flora of the Pedra de Fogo Formation traditionally has been correlated with the Chemnitz Petrified Forest in the Leukersdorf Formation of Germany (Rössler, 2006). Both palaeofloras show an abundance of marattialean tree ferns, and share at least three genera (Rössler, 2006; Iannuzzi et al., 2018). Originally considered to be of Kungurian age (Rössler, 2006), the Chemnitz Petrified Forest has recently been U–Pb dated as 291 ± 2 Ma (late Sakmarian; Luthardt et al., 2018). Other plant fossils from the Pedra de Fogo Formation, such as *Rachiphyllum*-like impressions, which are also found in the Saar–Nahe Basin of Germany, are instead suggestive of an earliest Permian age for this unit (Iannuzzi & Langer, 2014; Iannuzzi et al., 2018).

The recognition of the genus *Captorhinikos* in the Parnaíba Basin could be a source of potential stratigraphic information for the Pedra de Fogo Formation. *Captorhinikos chozaensis* is found in the middle Clear Fork Formation of Texas and the Hennessey Formation in Oklahoma (Jung, 2018), both of mid-Kungurian age. *Captorhinikos valensis* is found in the middle Clear Fork Formation of Texas, a unit also considered to be of mid-Kungurian age (Nelson, Hook & Chaney, 2013; Modesto, Lamb & Reisz, 2014; Lucas, 2017). However, the presence of this species in the Bally Mountains of Oklahoma raises the possibility that it has a longer stratigraphic range if that Oklahoman locality is correlated to the nearby Richards Spur site (LeBlanc et al., 2015), which has a U–Pb age of 289 ± 0.68 Ma (early Artinskian; Woodhead et al., 2010). Thus, the presence of *Captorhinikos* sp. does not offer a more precise age estimation for the Pedra de Fogo Formation than that previously inferred from other taxa recorded in that unit, but an improved correlation may be possible if material that can be definitively referred to *C. choazaensis* is eventually discovered.

The Pedra de Fogo beds share more tetrapods with North America than with other regions that yield Cisuralian continental faunas (Cisneros et al., 2015). The records of *Captorhinikos* sp. and an unidentified large moradisaurine add to the presence of two dvinosaur temnospondyls, *Timonya anneae* and *Procuhy nazariensis* (the latter being a trimerorhachid dvinosaur), in this formation (Cisneros et al., 2015), raising the total to four tetrapod species with North American counterparts. Although Cisuralian tetrapod-bearing rocks are known in Europe (Falconnet, 2014; Martens et al., 2005), South America (Piñeiro et al., 2011) and southern Africa (Warren et al., 2001; Rubidge, 2005), no dvinosaurs or captorhinids are recorded elsewhere at this time. Other tetrapods found in the Pedra de Fogo Formation include *Prionosuchus plummeri* (a long-snouted platyoposaurine temnospondyl) and at least one rhinesuchid. During the Cisuralian, platyoposaurines are only known in the Saar–Nahe Basin of Germany, whereas rhinesuchids are absent elsewhere, being recorded in other Gondwanan faunas starting in the Guadalupian (Marsicano et al., 2017; Eltink, Schoch & Langer, 2019). Together with these observations, the new captorhinid material reinforces the similarities of the Parnaíba Permian tetrapod assemblage with contemporary North American faunas. This hypothesis, however, should be taken with caution because the North American faunas have a long history of collection and sampling and are more extensively studied than their European counterparts. Information on other early Permian continental tetrapods from Gondwana likewise is scarce at this time (Warren et al., 2001).

**Figure 5:**
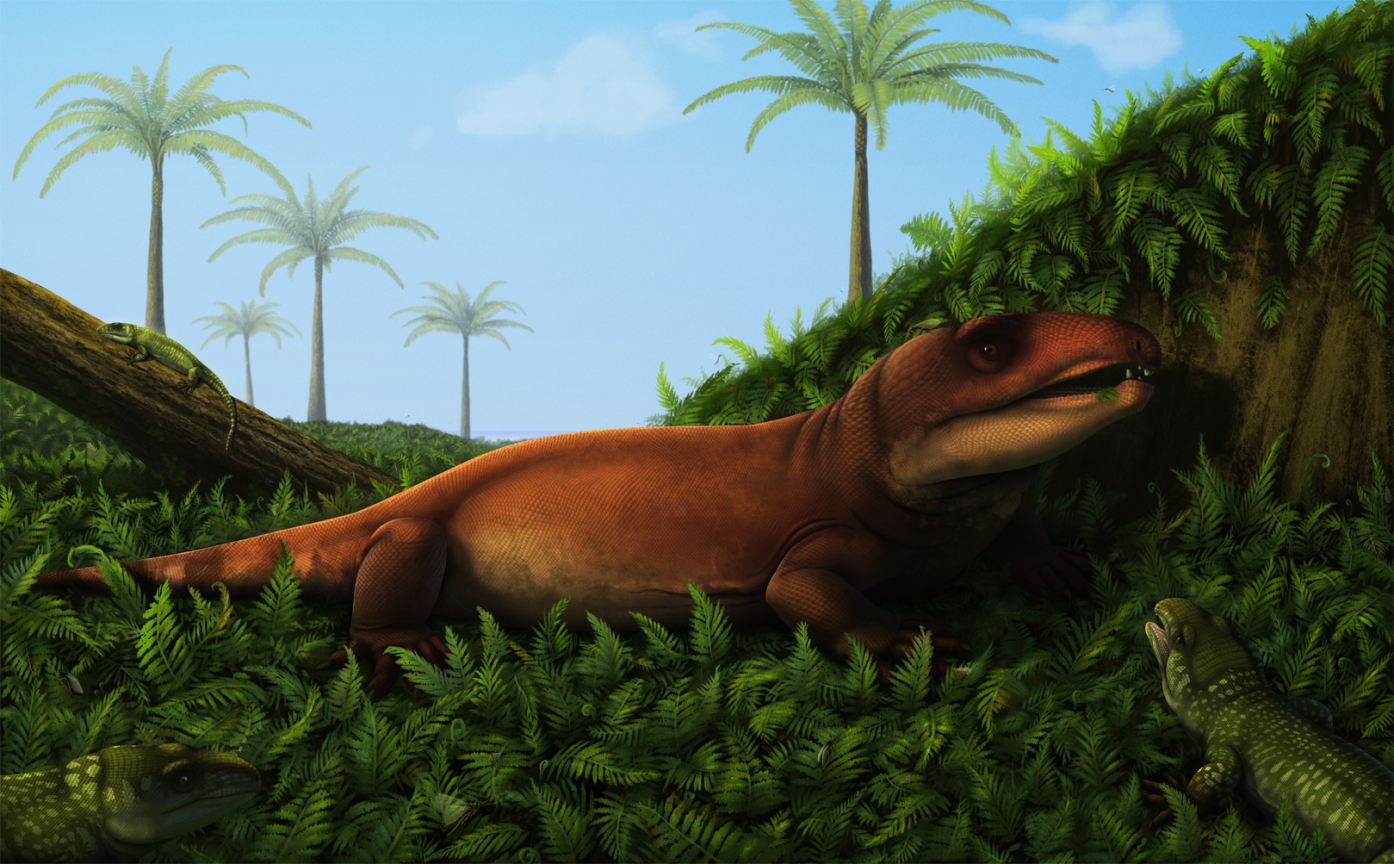
Reconstruction of the terrestrial palaeoenvironment in Piauí during the Cisuralian. Captorhinids represented: a probable moradisaurine (larger form) and *Captorhinikos* sp. (smaller form). Artwork by Vitor Silva.

Prior to the discovery of captorhinids, most of the Pedra de Fogo vertebrates were regarded as aquatic. These include marine forms (mostly chondrichthyans and bony fishes) found at sites closer to the depocenter of the basin in the states of Tocantins and southern Maranhão, and fresh-water forms (bony fishes and temnospondyls) in the east of the basin, in the Teresina region of Piauí. The latter area has been interpreted as an alkaline lacustrine/wetland system (Cisneros et al., 2015) that was bordered by plains inhabited by a rich flora dominated by arborescent plants (Da Conceição et al., 2016; Da Conceição, Cisneros & Iannuzzi, 2016; Iannuzzi et al., 2018). Captorhinids represent the first record of exclusively terrestrial tetrapods in the Pedra de Fogo Formation. These herbivorous reptiles doubtlessly made use of the rich floral resources that were available in an environment that appears to have been mostly humid, punctuated by multi-year dry events (Iannuzzi et al., 2018) (Fig. 5).

## Conclusions

At least two captorhinid morphotypes can be recognised in the Pedra de Fogo Formation: the smaller *Captorhinikos* sp., previously known from the southern USA, and a larger, unidentified moradisaurine that could represent a new taxon. A skull roof is regarded as Captorhinidae indet. Although it displays similarities to *Captorhinus*, the specimen is too incomplete to assign to a particular genus with any confidence. The presence of these taxa, including a genus previously known from Texas and Oklahoma, reinforces the biogeographical relationships of the Parnaíba Basin continental fauna with North American assemblages in the early Permian. Captorhinid reptiles played an important role as primary consumers in terrestrial ecosystems and their record in the Parnaíba Basin provides a more complete picture of this ancient biome in western Gondwana.

## Supplemental Information

10.7717/peerj.8719/supp-1 Supplemental Information 1 List of Characters.

10.7717/peerj.8719/supp-2 Supplemental Information 2 Data matrix employed in the analysis. Conservative Nexus file readable in *Mesquite* or equivalent programme.

10.7717/peerj.8719/supp-3 Supplemental Information 3 Implied weighting trees. Trees generated by implied weighting analysis (Newick format).

10.7717/peerj.8719/supp-4 Supplemental Information 4 Unweighted trees. Trees generated by unweighted analysis (Newick format).

## Institutional Abbreviations

MAP: (formerly UFPI), Museu de Arqueologia e Paleontologia, Universidade Federal do Piauí, Teresina, Brazil
PB: Parnaíba Basin locality (Museu de Arqueologia e Paleontologia catalogue)

## Additional Information and Declarations

### Field Study Permissions

The following information was supplied relating to field study approvals (i.e. approving body and any reference numbers): Fieldwork was authorised by Agencia Nacional de Mineração through collecting request 000.180/2016.

### Data Availability

The following information was supplied regarding data availability: The data matrix employed in the phylogenetic analysis, saved in conservative nexus format, is available as a Supplemental File. It contains a character list, explaining new characters and character modifications. Specimens described in the manuscript are stored at Museu de Arqueologia e Paleontologia, Universidade Federal do Piauí, Teresina, Brazil. Specimen accession numbers: MAP-PV014, MAP-PV015, MAP-PV664 and MAP-PV855.
