# Supplementary material for: Captorhinid reptiles from the lower Permian Pedra de Fogo Formation, Piauí, Brazil: the earliest herbivorous tetrapods in Gondwana
Source: PeerJ. 2020 Mar 6;8:e8719. doi: 10.7717/peerj.8719 (PMC7061909; doi:10.7717/peerj.8719)
Supplement: Supplemental Information 1 [file peerj-08-8719-s001.pdf]

Captorhinid reptiles from the lower Permian Pedra de Fogo Formation, Piauí, Brazil: the earliest herbivorous tetrapods in Gondwana

by JUAN C. CISNEROS[1], KENNETH D. ANGIELCZYK[2], CHRISTIAN F. KAMMERER[3], ROGER M. H. SMITH[4], JÖRG FRÖBISCH[5], CLAUDIA A. MARSICANO[6] and MARTHA RICHTER[7]

[1]Museu de Arqueologia e Paleontologia, Universidade Federal do Piauí, 64049-550 Teresina, Brazil; e-mail: juan.cisneros@ufpi.edu.br

[2]Negaunee Integrative Research Center, Field Museum of Natural History, 1400 South Lake Shore Drive, Chicago, Illinois 60605, U.S.A.; e-mail: kangielczyk@fieldmuseum.org

[3]North Carolina Museum of Natural Sciences, 11 West Jones Street, Raleigh, NC 27601-1029; e-mail: christian.kammerer@naturalsciences.org

[4]Department of Karoo Palaeontology, Iziko South African Museum, PO Box 61, Cape Town 8000, South Africa and Evolutionary Studies Institute, University of Witwatersrand, Private Bag 3, 2050 Johannesburg, South Africa; e-mail: rsmith@iziko.org.za

[5]Museum für Naturkunde, Leibniz - Institut für Evolutions- und Biodiversitätsforschung, Invalidenstr. 43, D-10115 Berlin, Germany and Institut für Biologie, Humboldt-Universität zu Berlin, Invalidenstr. 42, D-10115 Berlin, Germany; e-mail: joerg.froebisch@mfn-berlin.de

[6]Departamento de Cs. Geológicas, FCEN, Universidad de Buenos Aires, IDEAN-CONICET, C1428EHA Ciudad Autónoma de Buenos Aires, Argentina; e-mail: claumar@gl.fcen.uba.ar

[7]Earth Sciences Department, Natural History Museum, Cromwell Road, London SW7 5BD, UK; e-mail: m.richter@nhm.ac.uk

LIST OF CHARACTERS

New characters and character modifications

The character list is an amended version from Modesto et al. 2018. Codding changes are commented within the nexus file. We have avoided using the term "ogival" dentition, originally employed for labiolingually compressed, chisel-like crowns (character [10:3] in Modesto et al. 2018) that are present in *Captorhinus aguti* and *C. magnus*. By definition, an "ogive" is also a curved surface with a tapered apex, a geometry that also describes the bulbous teeth with conical apexes seen in forms such as *Labidosaurikos*, *Moradisaurus*, *Opisthodontosaurus* and *Captorhinikos*. For clarity, and in order to represent other states, we have replaced character 10 from Modesto et al. (2018) with two new characters: (10) Bulbous teeth on marginal dentition [0]

absent; [1] present; and (11) Marginal dentition crown morphology: [0] conical, recurved, [1] conical, not recurved, [2] labiolingually compressed, chisel-like.

Character (30), originally (29) in Modesto et al. 2018, has been redefined. Sculpturing: [0] light, with tiny pits and furrows, ridges low or absent; [1] moderate, with only small honeycombing pits or grooves; [2] coarse, with pits and grooves with notably larger, randomly positioned pits on posterior skull table. Character (62), was also redefined. Mandibular ramus: lateral shelf absent or weak [0]; lateral shelf well developed below coronoid process [1].

Four new characters relative to mandibular features have been added. Character (77): Dentary lingual shelf [0] absent; [1] present. The derived condition is usually associated to multiple tooth-rows. Character (78): diastema [0] absent or [0] present. Character (79) jaw sculpturing: [0] weak or absent; [1] well developed, covering most of the jaw; [2] well developed on the posterior half of the jaw, weak anteriorly. Character (80) sigmoideal step on lateral surface of posterior portion of the jaw: [0] absent; [1] present.

Skull roof

(1) Premaxilla: ventral margin aligned anteroposteriorly in lateral view [0]; ventral margin aligned anteroventrally in lateral view [1].
(2) Premaxilla: alary process absent [0]; alary process present on posterodorsal process [1].
(3) Premaxillary dentition: first tooth relatively small relative to maxillary caniniform [0]; subequal to maxillary caniniform [1]. In taxa lacking maxillary caniniforms, state 1 applies when the first premaxillary tooth is the largest marginal tooth present.
(4) Maxilla: relatively straight [0]; posterior end flexed laterally [1].
(5) Maxilla: posterior-most tooth positioned at level of posterior margin of orbit [0] or positioned more anteriorly [1].
(6) Maxillary dentition: tooth stations number 30 teeth or more [0]; teeth number 18–26 [1]; teeth number 14–17 [2]; teeth number 13 or less [3]. For multiple-rowed taxa, only those teeth with unobstructed profiles when viewed laterally are considered.
(7) Teeth on maxillary dental field: none [0]; less than 40 [1]; 40 or more [2].
(8) Maxillary caniniform teeth: present [0]; absent [1].
(9) Number of tooth rows in the upper jaw: one [0]; two to four [1]; five [2]; six or more [3].
(10) Bulbous teeth on marginal dentition: absent [0]; present [1].
(11) Marginal dentition crown morphology: conical, recurved [0]; conical, not recurved [1]; labiolingually compressed, chisel-like [2].
(12) Maxilla: double row of teeth extend far anteriorly on tooth row absent [0]; double row of teeth extend far anteriorly on tooth row present [1].
(13) Dental tooth wear: absent [0]; present, modest [1]; present, saddle shaped [2].
(14) Lacrimal: suture with jugal small [0]; well developed [1].
(15) Nasolacrimal suture: straight [0]; interdigitating [1].
(16) Snout: broad, equal to or greater than 35% of skull length [0]; narrow, equal to or less than 25% of skull length [1].
(17) Antorbital/cheek region: mainly formed by lacrimal and prefrontal [0]; mostly formed by lacrimal due to dorsal expansion of the bone [1].

(18) Prefrontal: prefrontal-nasal suture shorter than lacrimal-nasal suture [0]; prefrontal-nasal suture longer than lacrimal-nasal suture [1].
(19) Frontal: anterior process short, less than 40% of the frontal sagittal length [0]; anterior process long, approximately 55% of the frontal sagittal length [1].
(20) Jugal: subtemporal process dorsoventrally low, equal to or less than 25% of skull height through orbital midpoint [0]; subtemporal process dorsoventrally deep, at least 40% of skull height through orbit [1].
(21) Jugal: anterior extent ends posterior to anterior orbital margin [0]; anterior extent reaches beyond anterior orbital margin [1].
(22) Jugal: postorbital extent shorter than remaining anterior extent [0]; postorbital extent equal or longer [1].
(23) Quadratojugal: anteroposteriorly elongate [0]; short, not extending anteriorly beyond midpoint of postorbital margin [1].
(24) Quadratojugal: acuminate anteriorly [0]; square-tipped anteriorly [1].
(25) Quadratojugal: expanded dorsally [0]; posteriorly straight or decreasing in height [1].
(26) Quadratojugal: maximum height approximately equal to one-third or less that of squamosal [0]; nearly equal to half of squamosal height [1].
(27) Postorbital cheek: relatively straight [0]; moderately expanded laterally [1]; greatly expanded laterally [2].
(28) Parietal: distinct anterolateral process present that partially separates postfrontal and postorbital [0]; does not strongly project between postfrontal and postorbital [1].
(29) Pineal foramen: positioned at midpoint of interparietal suture [0]; anterior to midpoint of interparietal suture [1]; posterior to midpoint of interparietal suture [2].
(30) Sculpturing: light, with tiny pits and furrows, ridges low or absent [0]; moderate, with only small honeycombing pits or grooves [1]; coarse, with pits and grooves with notably larger, randomly positioned pits on posterior skull table [2].
(31) Supratemporal: obliquely oriented into anteromedial direction, thereby lying within a facet of the parietal [0]; positioned mediolaterally at the posterior edge of the parietal [1].
(32) Supratemporal: small, slender element [0]; large, contributing to skull table [1].
(33) Supratemporal-postparietal contact: tenuous or absent [0]; well developed [1].
(34) Postparietal: contacts mate dorsally only, postparietals separated slightly ventrally by supraoccipital [0]; contacts mate fully along height [1].
(35) Postparietal: transversely short with tabular present [0]; transversely elongate with tabular absent [1].
(36) Postparietal: contribution to skull table absent or forms only narrow edge [0]; contribution to skull table large, forming sculptured posterior portion of skull table [1].
(37) Skull table occipital margin: embayed bilaterally [0]; straight [1]; with single median embayment [2].

Palate

(38) Vomer: denticulated [0]; edentulous [1].
(39) Vomer-pterygoid contact: extensive, at least 50% median border of vomer [0]; short, no more than 33% median border of vomer [1].
(40) Palatine: denticulated [0]; edentulous [1].

(41) Jugal/ectopterygoid: ectopterygoid present and alary process absent [0]; ectopterygoid absent and alary process present, but no higher than the midpoint of the suborbital process of the jugal and distinct from the orbital margin [1]; ectopterygoid absent and alary process present and positioned dorsally on the medial surface of the jugal, flush with orbital margin [2].
(42) Dentition on palatal ramus of pterygoid: present [0]; greatly reduced or absent [1].
(43) Pterygoid: transverse flange dentition consists of shagreen of denticles [0]; transverse flange dentition consists of at least one row of functional teeth [1]; transverse flange dentition absent [2].
(44) Pterygoid: transverse flange broad-based and distinctly angular in ventral view [0]; transverse flange narrow and tongue-like in ventral view [1].
(45) Suborbital foramen: absent [0]; present [1]; anteroposteriorly elongate [2].

Braincase

(46) Parasphenoid: cultriform process extends anteriorly [0]; cultriform process extends slightly dorsally at roughly 15° to the basal plane [1]; cultriform process extends anterodorsally at more than 45° to the basal plane [2].
(47) Parasphenoid: edentulous [0]; denticulated [1].
(48) Parasphenoid: deep ventral groove between cristae ventrolateralis absent [0]; deep ventral groove between cristae ventrolateralis present [1].
(49) Parasphenoid: posterolateral wing narrow, meets narrow head of stapes [0]; wing broad, meets large head of stapes, in an elongate, nearly parasagittal suture [1].
(50) Opisthotic: paroccipital process long, extending near to medial edge of squamosal [0]; process short, extending only slightly beyond body of opisthotic [1].
(51) Paroccipital process: broad [0]; narrow [1].
(52) Stapes: distal process short [0]; distal process elongate [1].
(53) Supraoccipital: slopes anterodorsally [0]; aligned vertically or slopes posterodorsally [1].
(54) Supraoccipital: lateral ascending processes account for half or less height of bone [0]; lateral ascending processes account two-thirds or more height of the bone [1].
(55) Occipital condyle: at level of quadrate condyles [0]; immediately anterior to condyles [1].
(56) Exoccipital: lateral process on dorsal ramus absent [0]; lateral process present [1].

Mandible

(57) Dentary: Anterior dentary teeth: teeth uniformly small [0], multiple enlarged teeth present anteriorly [1]; single enlarged tooth present anteriorly [2].
(58) Dentary: first tooth oriented mainly vertically [0]; first tooth leans strongly anteriorly [1].
(59) Mandibular ramus: relatively straight in ventral view [0]; sigmoidal in ventral view [1].
(60) Mandibular ramus: narrow, 8% or less of total jaw length [0]; broad, no less than 14% of total jaw length [1].
(61) Mandibular ramus: posterior end rectilinear in lateral view [0] or acuminate in lateral view [1].
(62) Mandibular ramus: lateral shelf absent or weak [0]; lateral shelf well developed below coronoid process [1].
(63) Coronoid: anterior process short [0]; elongate [1].
(64) Meckelian foramen: small, anteroposterior length roughly 9% or less of total jaw length [0]; large, anteroposterior length greater than or equal to 14% of total jaw length [1].

(65) Coronoid: posterodorsal process slender, forms dorsal-most quarter of lateral wall of adductor fossa [0]; posterodorsal process deep, forms dorsal-most third of lateral wall of adductor fossa [1].
(66) Retroarticular process: absent [0]; present and broader transversely than long [1]; present and longer anteroposteriorly than broad [2].

Postcrania

(67) Dorsal neural arches: narrow [0]; lateral expansion present [1]; or exaggerated lateral expansion swelling present [2].
(68) Neural spines of dorsal vertebrae: height or shape alternation absent [0]; height or shape alternation present [1].
(69) Anterior dorsal centra: strongly ventrolaterally constricted [0]; not strongly ventrolaterally constricted [1].
(70) Sacral ribs: first and second sacral rib roughly equal in size [0]; first sacral rib larger than second rib [1].
(71) Ilium: iliac blade expanded dorsally [0]; iliac blade narrow dorsally [1].
(72) Stylo- and zeugopodium: shaft slender and heads only moderately expanded [0]; shaft massive and with proximal and distal heads significantly expanded, resulting in an overall stout impression [1].
(73) Humerus: supinator process parallel to shaft [0]; supinator process absent [1]. Modified slightly from Modesto *et al*. (2014).
(74) Manus and pes: elements long and slender [0]; elements short and broad [1].
(75) Fourth metatarsal: less than half the length of tibia [0]; more than half the length of tibia [1].
(76) First metacarpal: more than half the length of fourth metacarpal [0]; less than half the length of fourth metacarpal [1].
(77) Dentary lingual shelf: absent [0]; or present [1].
(78) Diastema: absent [0]; or present [0].
(79) Jaw sculpturing: weak or absent [0]; well developed, covering most of the jaw [1]; well developed, but more prominent on the posterior half of the jaw [2].
(80) Sigmoidal step on lateral surface of posterior portion of the jaw: absent [0]; present [1].

REFERENCES:

MODESTO, S. P., SCOTT, D., and REISZ, R. R. 2018. A new small captorhinid reptile from the lower Permian of Oklahoma and resource partitioning among small captorhinids in the Richards Spur fauna. *Papers in Palaeontology*, **4**, 293–307.
